# An 11-Year (2012–2022) review of *Journal of Athletic Training* publication study designs and sample sizes

**DOI:** 10.1186/s12874-025-02728-6

**Published:** 2025-12-30

**Authors:** Zachary K. Winkelmann, Samantha E. Scarneo-Miller, Emily C. Smith, Ryan M. Argetsinger, Lindsey E. Eberman

**Affiliations:** 1 Department of Exercise Science, University of South Carolina, Columbia, SC USA; 2 Elmer College of Health Sciences, Old Dominion University, Norfolk, VA USA; 3 Department of Applied Medicine and Rehabilitation, Indiana State University, Terre Haute, IN USA

**Keywords:** Qualitative, Quantitative, Methodology

## Abstract

**Background:**

Research findings must be representative by creating a sample of individuals, ensuring the results can be generalized and applicable to a larger population, which has historically been guided by a power analysis. However, the varied research design methods require a unique approach to sampling and a formula for recruitment and size. Therefore, the purpose of this study was to analyze historical data from published manuscripts in the *Journal of Athletic Training (JAT)* relative to study design and sample sizes. A secondary purpose was to further explore metrics for survey-based research.

**Methods:**

This descriptive analysis explored 1267 publications in each issue of the *JAT* from January 2012 (Volume 47) to December 2022 (Volume 57). We extracted publications from the *JAT* website. Every article was entered into a spreadsheet (year of publication, publication title) and data specific to the study design and sample size were used for analysis. For studies that were coded as survey-based research, access, response, and completion rates were completed, and topic area and use of a power analysis were extracted. Data were analyzed using measures of central tendency (mean, median, range).

**Results:**

Of the 1267 published studies, the most frequent design was cross-sectional (394, 31.1%). In total, 1080 publications (85.2%) were not survey-based, with a median sample size of 34 participants, while 187 publications (14.8%) were survey-based, with a median sample size of 429. Among those surveys, most were cross-sectional (*n* = 151/187, 80.8%), with 80.7% (*n* = 151/187) reporting the number initially recruited and 50.8% (*n* = 95/187) reporting the number of surveys started. The survey publications reported recruiting an average of 4453 potential participants (median = 2500; min = 101, max = 48752), with 985 participants starting the study (median = 816, min = 57, max = 7067), and a final sample size of 819 (median = 429; min = 17, max = 13002). The grand mean access rate was 22.1%, the grand mean response rate was 18.4%, and the grand mean completion rate was 83.1%.

**Conclusion:**

Researchers and reviewers can use these trends to guide authorship and review processes for athletic training research. However, sampling strategies should be consistent with the research question, which may lead to deviations from these reported trends.

## Background

The *Journal of Athletic Training (JAT)* serves as one of the leading platforms for scholarly research in the field of athletic training and sports medicine. At its core, the *JAT*’s mission is to “advance the scientific understanding and clinical practice of athletic training and sports medicine” and “provide the evidence to aid clinicians in performing evidence-based practice” [1]. Since its acceptance into MEDLINE in March 2007, the *JAT* has achieved significant recognition, including being ranked a top-ten sports science journal by Thomson Reuters Web of Science [2]. The transition in 2015 to a digital format and an increase in publication frequency from 4–6 issues per volume to 12 issues per volume reflect the growing volume of research in this field.

The journal’s peer review process for accepting high-quality research tasks requires peer reviewers and editors to identify the manuscript’s importance to athletic training, overall content, overall presentation, and priority of publication compared to other papers in the field. In 2018, the *JAT* leadership set a strategic goal to enhance its impact factor by adopting a more selective publication strategy, focusing on the top 20% of submissions that demonstrated significant clinical applicability and potential for advancing the field [1]. Areas that can influence the priority of the publication to the field include the study design and sample size. The increased rigor of the *JAT* resulted in an explicitly communicated preference for publication in “studies that employ robust research designs and focus on patient-oriented clinical and translational research” [1]. Previous research has identified that other scholarly outlets, such as *BMJ*,* Lancet*, and *Annals of Internal Medicine*, manuscripts were more likely to be accepted if they were a randomized controlled trial (RCT) and had larger sample sizes [3]. Despite these findings, other journals, such as *Journal of Bone and Joint Surgery* and *Clinical Orthopedics and Related Research* identified that in the 33 level 2 (RCTs) manuscripts published, only 9% described sample calculations and 48% of the studies lacked the power necessary to detect a large effect size [4]. These findings bring to light the discrepancies in publishing high-quality research with respect to study design and sample sizes.

The manuscripts published by the *JAT* focus on patient care, including research completed on athletes and other physically active populations, as well as professional concerns, including research on athletic trainers, athletic training students, and other stakeholders. The target population for these studies influences the potential pool of participants. Despite these advancements, there remains a gap in empirical data regarding the preferred study design categories and sample sizes that align with the *JAT’s* publication criteria. This study aims to analyze historical data (2012–2022) from manuscripts published in the *JAT* to assess trends in study design and sample sizes. Additionally, we seek to investigate survey-based research metrics specific to these publications to provide insights into best practices for editors, reviewers, and researchers in this domain.

## Methods

### Study design

We used a retrospective descriptive analysis of previously published manuscripts to answer our research question. We systematically searched for, extracted, and reviewed eligible manuscripts from one sports medicine journal: the *JAT*, which adheres to a rigorous peer review process and is considered one of the most influential for athletic training-related searches. As this study is non-human subject research, the project did not require approval from the ethics review board (Clinical trial number: not applicable).

## Procedures

To accomplish the research aims, the researchers created a database of the studies from the *JAT*. The primary investigator (ZKW) trained two research assistants (KA, AR) to extract studies from the online *JAT* website (https://meridian.allenpress.com/jat) published in the monthly or bi-monthly issues from January 2012 (Volume 47) to December 2022 (Volume 57). The rationale for selecting the previous 11 years was due to leadership changes within the journal. Dr. Craig Denegar served as Editor-in-Chief from 2012 to 2017, followed by Dr. Jay Hertel, who assumed the role in 2018 when the new priorities for the journal were set [1]. We began data analysis in March 2023, thus establishing the boundaries for inclusion between 2012 and 2022.

To create our database of the articles, the research assistants (KA, AR) extracted the publication title, the month and year of publication, the study design category reported in the manuscript, and sample size information in the publication. In addition, a hyperlink to the online article was placed in the spreadsheet. Depending on the study design category, modifications were made to the reported information. For example, all studies were further categorized as survey or non-survey research. If it were a survey, the number of invitations to participate sent and started per study was extracted. Studies with mixed methods, epidemiological, systematic reviews, and/or meta-analyses also had specific criteria based on their samples to align with their reporting structure. For example, when reviewing epidemiological studies, data collected in the spreadsheets reported the number of athletes, injuries, and exposures. In meta-analyses and systematic reviews, the spreadsheet included classification and number of studies included for the criteria. Data from mixed methods studies included the number of invitations to the survey, the number of surveys started, the qualitative sample size, and the final sample number in the study. After completing the extraction of all published studies from 2012 to 2022, the primary author (ZKW) cross-analyzed 20 randomly selected articles to ensure the process for data extraction was followed appropriately by the research assistants.

After the database was established, our research team, consisting of 5 individuals (ZKW, SESM, ECS, RMA, LEE), divided the total number of studies (*n* = 1267) to perform a secondary analysis of the reported information by verifying the original manuscript. The primary author (ZKW) trained each member to review a publication and extract data from both the abstract and methods, as information could be shared differently, in multiple places, or limited to a specific portion of the paper. The training was initially conducted as a one-hour, online synchronous session where we explored the data spreadsheet, definitions, and completed an exemplar article together. The research team had ongoing dialogue throughout the data extraction process. As a note, many published articles had unique study design names to correspond with their methodology. As a result, the researchers streamlined and coded the study designs as one of 17 options (Table 1) that closely aligned with the *JAT* Author Guidelines [5]. The data extraction occurred between March to June 2023.

**Table 1:** Study design classifications

Study Design Type	Included in JAT 2020 Author Guidelines (yes/no)
Case Series	Y
Case Study/Case Report	Y
Case-Control	Y
Cohort	Y
Commentaries	Y
Controlled Laboratory	Y
Crossover	Y
Cross-Sectional	Y
Delphi	N
Descriptive Laboratory	Y
Epidemiology	N
Implementation Science	N
Meta-Analysis and Systematic Review	Y
Mixed Methods	N
Observational	N
Qualitative	Y
Randomized-Controlled Trials	Y

Finally, once the researchers had compiled all the information, we performed an additional verification check to cross-analyze each other’s work, followed by a final analysis by the primary investigator (ZKW). A meeting was scheduled to discuss all manuscripts flagged during the extraction or cross-analysis process as conflicting or missing information in July 2023. We reviewed 63 conflicted articles (4.97% of the total sample) and took a consensus vote (3/5) on the final coding decision.

### Data analysis

Data were then transferred into a spreadsheet for descriptive analysis through Statistical Package for Social Sciences (SPSS Version 28, Armonk, NY) between August and September 2023. The analysis included (1) an exploration of the number of studies published per year, (2) the number per study design category published over the 11 years, (3) the sample size of published manuscripts by study design category, and when applicable, (4) exploring the number of studies that were survey versus non-survey and their respective sample sizes. For studies that were coded as survey-based research, we identified (1) if a power analysis was performed, (2) the content area and population of the study, and finally, (3) calculated access, response, and completion rates, when applicable. We operationally defined the rates as follows:

- Access rate: Number of surveys started by number of emails sent (total number in sample group).
- Response rate: Number of surveys completed divided by number of emails sent (total number in sample group).
- Completion rate: Number of surveys completed divided by the number of respondents who started/opened the survey.

## Results

### Study design category

Table 2 presents a summary of the annual publication of studies and the number of survey-based research manuscripts. On average, the *JAT* publishes approximately 115 manuscripts per year, with around 17 of these being survey-based studies. Among the 1,267 studies published in the dataset, the predominant research design was cross-sectional (*n* = 394, 31.1%), followed by commentaries (*n* = 130, 10.3%).

**Table 2:** By-Year analysis of published JAT manuscripts (2012–2022)

Year	Total Manuscripts Published	Total Survey-Based Research Manuscripts Published
2012	65, 5.1%	6, 9.2%
2013	78, 6.2%	11, 14.1%
2014	73, 5.8%	11, 15.1%
2015	131, 10.3%	17, 13.0%
2016	91, 7.2%	11, 12.1%
2017	117, 9.2%	12, 10.3%
2018	130, 10.3%	15, 11.5%
2019	151, 11.9%	29, 19.2%
2020	150, 11.8%	27, 18.0%
2021	161, 12.7%	26, 16.1%
2022	120, 9.5%	22, 18.3%
Totals	1267, 100%	187, 100%

Table 3 details the distribution of study designs and sample sizes for the published research. Notably, no manuscripts in the dataset fell under the quality improvement report category, which is listed as a design option in the *JAT* author guidelines. Table 4 compares survey-based and non-survey-based publications, showing that 1,080 publications (85.2%) were categorized as non-survey research. In comparison, 187 publications (14.8%) were identified as survey research, irrespective of their specific design category.

**Table 3:** Sample sizes by design category of JAT manuscripts (2012–2022)

Design Category	Number of Published Studies (*n* = 1267)	Range and Median Sample Size	Number of Surveys by Design (*n* = 187)
Case Series	19, 1.5%	Min = 2, Max = 1534 Median = 24	0, 0%
Case Study/Report	11, 0.9%	Min = 1, Max = 1 Median = 1	0, 0%
Case-Control	48, 3.8%	Min = 10, Max = 2696 Median = 40	2, 1.1%
Cohort	105, 8.3%	Min = 8, Max = 701,027 Median = 84	7, 3.7%
Commentaries	130, 10.3%	N/A	N/A
Controlled Laboratory	58, 4.6%	Min = 5, Max = 239 Median = 21	0, 0%
Crossover	72, 5.7%	Min = 4, Max = 51 Median = 17	0, 0%
Cross-Sectional	394, 31.1%	Min = 7, Max = 19,918 Median = 153	151, 80.8%
Delphi	3, 0.2%	Min = 8, Max = 17 Median = 16	0, 0%
Descriptive Laboratory	84, 6.6%	Min = 5, Max = 4045 Median = 35	1, 0.5%
Observational	18, 1.4%	Min = 6, Max = 71,302 Median = 241	0, 0%
Epidemiology	104, 8.2%	Min = 1606, Max = 35,581,036 AE median = 444,725	0, 0%
Implementation Science	2, 0.2%	Min = 62, Max = 1755 Median = 908	2, 1.1%
Meta-Analysis and Systematic Review	40, 3.2%	Min = 4, Max-=238 Median = 17 MA = 20, 50% SR = 20, 50%	0, 0%
Qualitative	91, 7.2%	Min = 6, Max = 513 Median = 19	4, 2.1%
Mixed Methods	19, 1.5%	Min = 17, Max = 10,553 Quan Median = 442 Min = 6, Max = 10,553 Qua Median = 22	19, 10.2%
Randomized-controlled trials	69, 5.4%	Min = 10, Max = 56,000 Median = 39	1, 0.5%

**Table 4:** Sample size and rates based on survey and Non-Survey research

Classification	Total Published	Sample Size	Access Rate	Response Rate	Completion Rate
Survey	187, 14.8%	Median = 429 Mean = 819 Min = 17 Max = 13,002	*n* = 88 Median = 21.5%	*n* = 151 Median = 20.4%	*n* = 95 Median = 81.6%
Non-Survey	1080, 85.2%	Median = 34 Mean = 1403 Min = 1 Max = 701,027	N/A	N/A	N/A

### Sample sizes

The variation in sample sizes by study design category is widely noted, with case reports having a median of 1 participant and observational studies having a median of 241 participants. The epidemiological studies (*n* = 104) had the largest sample size, with an average athlete exposure of 444,725. The median sample size of non-survey manuscripts was 34 participants. In contrast, the median sample size of the survey research publications was 429 participants (Table 4).

### Survey-Based research

Most publications categorized as surveys were cross-sectional designs (*n* = 151/187, 80.8%). The amount of survey research seems to be positively trending, with the average from 2012 to 2017 being 12 manuscripts annually compared to 26 from 2018 to 2022, as demonstrated in Table 1. Of the 187 survey-based studies, the majority (*n* = 105, 56.1%) studied practitioners or those in training, such as athletic trainers or athletic training students. In contrast, fewer studied patients/athletes (*n* = 42, 22.5%), athletic personnel such as coaches and administrators (*n* = 17, 9.1%), or other groups (*n* = 23, 12.3%) such as physicians, parents, or legislators. The surveys typically focused on healthcare competency or vitality of the profession. Only ten studies (5.3%) noted the authors’ use of a power analysis in justifying the sample size, with 50% of these (*n* = 5/10) focused on athletes or patients.

When exploring the data from 2012 to 2022, the survey-based studies showed the variance in the number of publications over time and the shifts in the access, response, and completion rate percentages over the 11 years. Interestingly, 80.7% (*n* = 151/187) of publications using survey methodology reported the data necessary to calculate a response rate. In comparison, 50.8% (*n* = 95/187) reported the data needed for a completion rate, and 47.1% (*n* = 88/187) reported the data necessary to calculate an access rate. On average, the survey publications recruited 4,453 potential participants (median = 2500; min = 101, max = 48752), with 985 participants starting the study (median = 816, min = 57, max = 7067), and a final sample size of 819 (median = 429; min = 17, max = 13002). The grand mean access rate was 22.1% (*n* = 985/4453; median = 21.5%), the grand mean response rate was 18.4% (*n* = 819/4453; median = 20.4%), and the grand mean completion rate was 83.1% (*n* = 819/985; median = 81.6%). Table 5 provides specific ranges and means of the participation rates of survey-based research by year, demonstrating a consistent 70–88% completion rate for survey-based studies.

**Table 5:** Descriptive statistics of survey-based studies from JAT manuscripts (2012–2022)

Year Published		Number of Studies Reporting Data	Range and Means of Participation Rates
2012	Access Rate	2	Min = 39.29, Max = 73.09 Mean = 56.19 ± 23.90
	Response Rate	6	Min = 23.76, Max = 72.36 Mean = 47.51 ± 19.68
	Completion Rate	2	Min = 74.55, Max = 89.55 Mean = 82.05 ± 10.61
2013	Access Rate	4	Min = 18.04, Max = 36.15 Mean = 25.94 ± 7.83
	Response Rate	11	Min = 10.01, Max = 82.59 Mean = 27.29 ± 19.22
	Completion Rate	4	Min = 75.53, Max = 100.00 Mean = 87.92 ± 10.57
2014	Access Rate	4	Min = 11.25, Max = 31.18 Mean = 25.01 ± 9.30
	Response Rate	6	Min = 7.74, Max = 72.60 Mean = 32.24 ± 21.69
	Completion Rate	5	Min = 34.67, Max = 91.92 Mean = 73.86 ± 23.61
2015	Access Rate	6	Min = 16.40, Max = 93.21 Mean = 42.99 ± 35.82
	Response Rate	15	Min = 10.87, Max = 70.92 Mean = 37.94 ± 19.32
	Completion Rate	7	Min = 55.98, Max = 95.72 Mean = 78.07 ± 13.91
2016	Access Rate	5	Min = 25.10, Max = 55.34 Mean = 36.15 ± 12.10
	Response Rate	10	Min = 15.40, Max = 53.40 Mean = 28.52 ± 11.76
	Completion Rate	5	Min = 75.30, Max = 99.40 Mean = 88.71 ± 9.95
2017	Access Rate	4	Min = 21.85, Max = 49.38 Mean = 34.87 ± 13.12
	Response Rate	9	Min = 12.63, Max = 54.13 Mean = 36.31 ± 14.75
	Completion Rate	4	Min = 79.41, Max = 91.89 Mean = 86.57 ± 6.11
2018	Access Rate	10	Min = 10.33, Max = 44.56 Mean = 26.26 ± 11.15
	Response Rate	13	Min = 9.20, Max = 39.38 Mean = 20.87 ± 10.22
	Completion Rate	10	Min = 61.28, Max = 98.63 Mean = 76.51 ± 14.07
2019	Access Rate	19	Min = 10.28, Max = 98.29 Mean = 33.50 ± 30.91
	Response Rate	26	Min = 2.09, Max = 98.29 Mean = 30.90 ± 30.36
	Completion Rate	20	Min = 60.59, Max = 100.00 Mean = 83.05 ± 13.16
2020	Access Rate	12	Min = 10.28. Max = 94.86 Mean = 21.74 ± 23.36
	Response Rate	19	Min = 6.84, Max = 90.95 Mean = 24.30 ± 23.19
	Completion Rate	14	Min = 53.92, Max = 99.32 Mean = 80.57 ± 13.37
2021	Access Rate	12	Min = 4.24, Max = 92.35 Mean = 34.17 ± 31.79
	Response Rate	19	Min = 3.11, Max = 93.43 Mean = 33.80 ± 33.44
	Completion Rate	13	Min = 34.40, Max = 97.43 Mean = 72.80 ± 18.50
2022	Access Rate	10	Min = 9.90, Max = 100.00 Mean = 31.08 ± 34.84
	Response Rate	17	Min = 5.00. Max = 100.00 Mean = 22.77 ± 23.03
	Completion Rate	11	Min = 12.89, Max = 100.00 Mean = 70.54 ± 25.66

## Discussion

This project aimed to explore the study design categories and sample sizes of published manuscripts in the *JAT.* Between 2018 and 2022, the number of published manuscripts (*n* = 712) was higher than those published in 2012 and 2017 (*n* = 555). Our data suggests that trends have emerged in the study design categories published within the journal, with 31% of all manuscripts published being cross-sectional studies.

### Overview of main findings

#### Study designs

Cross-sectional study designs allow researchers to collect relevant data quickly and efficiently while estimating the prevalence of disease/traits, attitudes, or knowledge of a sample at a given time [6]. Cross-sectional studies are also a helpful starting point when there is a lack of information on a specific topic. Therefore, it was unsurprising that most *JAT* publications were cross-sectional designs. However, the variability in data collection methods and representation may require greater standardized criteria for study classifications. Most (80.1%) of the 187 studies containing survey research were also cross-sectional designs. Using a cross-sectional design study for a questionnaire allows for the gathering of larger sample sizes rapidly [7].

Commentaries were the second most prevalent design type published by the *JAT*. Engagement in evidence-based practice is essential; documents published in the *JAT*, such as position statements, literature reviews, and editorials, allow clinicians to quickly review the best practices for specific topic areas. As a partner of the National Athletic Trainers’ Association (NATA), the *JAT* has published the position statements curated by the Pronouncements Committee since its inception in 2011; however, the first position statement on lightning safety was published a decade earlier in 2001, not in a peer-reviewed journal. Since 2018, the *JAT* has published six position statements throughout all domains of clinical practice. The results of our study suggest that the publication of these materials has been consistent and may serve as a valuable resource to the readership.

An interesting finding was the number of Level 1 and 2 research publications. In total, 40 systematic reviews or meta-analyses and 69 RCTs were published in this sample, accounting for 9.6% of all publications within the journal. Previous research explored similar outcomes by exploring Level I evidence published within five sports medicine journals [8]. Their data identified nonsurgical studies with therapeutic inventions as the most common Level I evidence publications in those journals [8]. This study compares multiple journals’ choices in publication for a higher level of evidence, but it also considers other levels that may provide significant and relevant information for readers. In reviewing our data, we found that 65.2% of RCTs were published between 2012 and 2018. Sports medicine research is in its infancy compared to other established research fields, such as cancer and smoking. With this only being the outset, there is a need to characterize the “problem” being investigated, which often results in the need for cross-sectional or observational study designs. As the field continues to evolve, we are hopeful of an increase in additional Level 1 and 2 research studies within athletic training. The importance of systematic reviews and meta-analyses in sports medicine and other areas of research has become increasingly necessary for evidence-based clinical decision-making; however, when evaluating premier orthopedic sports medicine journals, the rate of publications of Level 3–5 systematic reviews and meta-analyses has increased exponentially over the past decade, while Level 1–2 systematic reviews have been outpaced [9]. The critical importance of systematic reviews allows researchers to utilize compiled evidence as a basis for future studies and digestible literature for readers, which is why it is essential to maintain the quality of evidence. Thus, maintaining standards regarding scientific literature allows for clinical translation, justifiable investigation, and efficient practices. Moreover, RCTs provide a high level of evidence, which can be supported through their design, especially in sports medicine and athletic training; however, recent trends in utilizing cohort study models can be ideal for assessing the association among multiple characteristics and clinical outcomes that are necessary between the relationship of athletes and athletic trainers [10]. While RCTs provide strong internal validity, cohort studies match with an external validity that is highly relevant to clinical decision-making [11].

As the athletic training profession continues to evolve, there is a need for more implementation science. These studies provide valuable information on how and potentially why an implementation is successfully integrated into clinical practice. However, there are no guidelines for implementation science studies within the *JAT* author guidelines, which could lead authors to avoid publishing using this study design in the journal as they explore outlets. Translation research, dissemination studies, and implementation science require unique data presentation guidelines. As the profession moves from basic science and efficacy studies into effectiveness studies and practice through translation to practice and the community, we must advocate for guidance in curating scholarly contributions for clinicians on how to take and apply basic science findings.

### Sample sizes

Clinical studies, social statistics, and survey research commonly utilize nonprobability-based sampling methods [12, 13]. Due to this, it is essential for researchers to consider that although calculating sample size is important for the generalizability of the results, the estimate when using nonprobability sampling may be irrelevant, as seen with convenience sampling tends to generate nongeneralizable results and prevents statistical inference to larger populations [14]. Although most academic journals do not place limitations on sample sizes, both ends of the spectrum can produce significant discrepancies in results: insufficiently small sample sizes create challenges in the replication of results, potentially raising false negatives, while a larger sample size may lead to *p-values* less than the significance level, even if the effect is not for practical or clinical importance [15, 16].

In other healthcare professions, eight leading dental specialty journals were reviewed for their quality practices of RCT sample size determination. This study found that 29% (*n* = 121/413) of RCTs identified information to allow replication of sample size calculations, and found evidence that journal methodology involvement, multi-center settings, and time since publication were all significant predictors of adequate description of sample size assumptions [17]. These factors and considerations allow for researchers to have standardized practices for sample size collection while maintaining the efficacy of practices for publications in scientific literature journals and providing transparency to readers and fellow investigators, as well. Without proper representation of sample sizes and the calculations required to describe them, the potential for suboptimal reporting and evidence is a concern to be considered. A power analysis is used to justify a study’s sample size [18]. A power analysis is a proper approach for studies that will involve participants in a clinical or practical setting because of its ability to better inform investigators of a reliable effect [16, 19]. Power analyses are crucial for inferential and probability-based studies and help to limit Type I and Type II errors; however, descriptive statistics do not draw inferences from probability theory of a population being studied, but summarize the general investigation [12, 20]. Researchers must carefully decide on the sample size to best fit their primary research question, including using power versus precision. Power is often used for hypothesis testing (the number of participants required to reject the null hypothesis). In contrast, a precision approach focuses on the number of participants needed to meet a confidence interval repeatedly [21]. The findings of our study suggest that the typical statistical approach using a power analysis may not be relevant and meaningful for descriptive, cross-sectional, survey-based research.

Of the 1267 published studies reviewed in this research, approximately 15% (*n* = 187) of study designs were labeled survey-based regardless of design category. Data from publications in the *American Journal of Pharmaceutical Education* (*AJPE*) suggest that sample size should be decided on a variety of factors, such as population characteristics and study design, and should effectively focus on response representativeness, rather than entirely on response rate [22–24]. The *AJPE* also recommends authors to report the methods of estimating sample size and the impact of potential non-response bias [17].

According to Charan and Biswas, sample size formulas cannot be treated as a universal key for all research designs [25]. Multiple formulas have been created for specific study designs considering the quantitative and qualitative variables; however, the degree to which technology has enhanced the accessibility of research to the masses may influence recruitment procedures [25]. In survey research, a large sample is expected to be used to generalize the findings. In doing so, it is best practice for researchers to report how many people received an email and how many responded. On the contrary, laboratory-based research often uses fliers and word-of-mouth sampling to recruit participants; however, there is never an expectation to report how many potential participants saw those fliers or had a conversation, as it is not common practice to think about how many potential participants could have opted in. This is a critical issue where survey-based and laboratory-based research expectations differ. The *International Journal of Sports and Exercise Medicine* (*IJSM*) published a guideline of ethical standards for authors and investigators regarding sample size and several other recommendations [26, 27]. The *IJSM* recommends that minimal sample size be determined through adequate statistical power and should be considered alongside study procedures, while considering the scientific or clinical importance of the study [26, 27]. The *IJSM* recognized that smaller studies may not be unethical if there is a high importance practically and clinically, even if statistical significance is not fully met [26, 27]. This aligns well with our data, suggesting that professional concern research, often captured through survey-based studies, may be essential for the advancement of the profession.

When considering cross-sectional studies, for example, the validity of the population being represented in the sample is most necessary when determining the efficacy and significance of the study, including how the sample was calculated and whether it is representative of the studied population [20]. A power analysis may be unnecessary for studies using descriptive statistics where a formal hypothesis is not being tested [28]. We recommend that survey researchers explore the use of sample size calculators, such as those from Qualtrics (https://qualtrics.com/blog/calculating-sample-size) or Creative Research Systems (https://www.surveysystem.com/sscalc.htm). The calculators use confidence levels and margins of error to describe an ideal sample size from the total population [29]. This type of calculator is best suited for projects that measure a percentage or proportion of the sample population with some characteristics, or compare proportions with another group [29].

Sample sizes can vary depending on the variables and design of the study and if the sampling technique is complicated and costly [30]. For research in the area of professional concerns, meaning examining behaviors, knowledge, or trends of athletic trainers or athletic training students, researchers have historically used e-mail services from the NATA or the Board of Certification for the Athletic Trainer (BOC). The associated cost of sending e-mails through these services and the increasing need for participant incentives make survey research costly to achieve the “desired” sample size. The sample specifications allow you to recruit only those who have opted to share their information, which varies between 20,000 and 32,000 individuals. From there, the researcher can provide specific criteria for their sample, including certification date, professional setting, and gender identity, among other characteristics. However, there are also limits to this sample in terms of the broad profession and reaching members from every facet. There are discounted rates for members of the NATA and those who are BOC-approved providers, but the costs for both organizations range from 9 to 60 cents per e-mail address. There are additional fees for the length of data collection. For example, if a researcher wanted to conduct a descriptive study of all BOC-credentialed athletic trainers who have opted into the database, it would cost approximately $2,880.00 ($0.09 per name based on discounted prices). In contrast, a study of the entire database from the NATA (approximately 23,000 e-mails) would cost a member around $5,050 ($0.20 per name with an 8-week data collection window) [31]. These costs do not equal the final number of people participating in the survey, which is why the access rate is critical in survey-based research. The use of the survey research service from the NATA provided us with a look into data collected, rather than published, from profession-specific recruitment methods. Table 6 provides an overview of associated costs and survey service data from 2017 to 2024. Based on the data provided by the NATA Survey Research Service, the average sample size based solely on access rate would be 322 participants, which varies drastically from the average sample size of 819 participants published in *JAT*. This number does not account for the participants who do not wish to participate, do not complete the study, and/or are removed from the analysis. However, the data provided by the NATA Survey Research Service also supports the notion that more than 50 survey-based projects are conducted annually. Yet, the *JAT* is publishing fewer than 20 annually.

**Table 6:** NATA survey service data and approximate costs

Years	Number of Projects sent by NATA Survey Service	Average Number of Emails Sent to Participant Pool	Potential Expected Costs for NATA members conducting an 8-week data collection	Mean Access Rate	Potential Mean Sample Size
2017–2018	32	3,355	$1,121	unknown	N/A
2018–2019	48	3,980	$1,246	13%	517
2019–2020	92	3,117	$1,073	13%	405
2020–2021	74	2,770	$1,004	9%	249
2021–2022	57	3,980	$1,246	8%	318
2022–2023	67	3,256	$1,101	10%	326
Average	62	3,409	$1,131	11%	363

Similarly, a power analysis does not calculate sample sizes in qualitative research and does not display the importance of qualitative research comprehensibly or at all [32]. These methods allow a deeper understanding of the experiences of peers or other stakeholders. Qualitative research utilizes interview techniques to describe environments, experiences, culture, and phenomena. There is no set sample size guideline for qualitative research studies, as interviews typically cease when saturation is met in the interview transcription [33]. Inductive thematic saturation occurs when the collected and analyzed data no longer generate new themes or ideas [34]. In our findings, qualitative research had a median of 19 participants with a maximum of 513 participants. The sample size maximum was from a qualitative study that used a survey-based methodology to collect written reflections instead of an interview-based data collection approach. Based on these findings, we recommend that peer reviewers recognize that most qualitative studies have between 8 and 20 participants [35]. This is considered an on-par sample size for qualitative research and should not be reflected as “small” in the review process.

Findings from our study support the capability of well-developed survey research when paired with cross-sectional study designs.

However, for the same manuscripts that utilized cross-sectional studies and surveys, initial sample sizes were reported in 80.7% of publications, while 50.8% of studies reported the number of surveys started. This indicates that many publications reported the initial number of recruited emails or participants, but nearly half excluded or did not report the number of surveys started. It is essential to report access and response rates along with the completion rates, as this will produce a clear image of the data reported. Response rates vary by the type of research being conducted, especially depending on the sample population and factors that may influence response, such as the format of survey collection [36]. When evaluating trends in research for recommendations among response rate guidelines, studies show that multiple medical journals suggest researchers aim for a response rate of 60% [37]. Asch et al. sampled 20 medical journals to find response rates varied depending on the subject studied and techniques used: published surveys of physicians’ average response rate was 54%, while non-physicians averaged a mean response rate of 68% [37]. In comparison, education-related studies yielded a 44% response rate [36]. Other studies have found that online surveys yielded lower response rates than all other modes due to reasons such as the legitimacy of the survey and the researcher’s effort [38, 39].

When surveys are sent to more participants, the yield of a higher response rate is not guaranteed. It may not be necessary depending on sample populations, so it is recommended to develop a refined population to which to send surveys [36]. Moreover, response rates indicate the extent of non-respondent bias, which is why researchers and academic journals should assess bias, rather than response rate thresholds among target samples [37]. It should also be recommended that a survey’s response rate acts as an indirect indication of the non-respondent bias, forcing investigators and journal editors to focus more on assessments of bias and less on response rate thresholds [36]. The survey publications reviewed displayed a grand mean access rate of 22.1%, a grand mean response rate of 18.4%, and a grand mean completion rate of 83.1%. Authors must take time to report these rates accurately in both abstracts and results to provide the most accurate and generalizable data. However, historical data on response rates are often guided by outdated procedures, such as mail-in surveys, with the illogical conclusion that researchers should aim for a 60% response rate or that to achieve power in survey research, the researcher should have ten participants per question in the survey [40, 41]. In a review of peer healthcare profession scholarly outlets, we explored explicit journal expectations for response rates. While one journal reported an “expected” response rate to achieve generalizable findings [22], much of this data is not publicly available and can be wide-ranging. Despite the use of a poor response rate as justification for rejection decisions in athletic training, the JAT currently has no publicly documented expectation for access, completion, or response rates. The data also illustrate a subtle and gradual increase in access, response, and completion rates (number of participants throughout the studies) from the publication years of 2012 to 2019. Means of access (~ 22–56%), response (~ 21–48%), and completion (73–89%) rates remained relatively consistent over the years despite increases in the number of participants reported. The access, response, and completion rate data results provide insight into the importance of stating and quantifying sample sizes throughout research, especially when using a smaller sample size that cannot be extrapolated to further data sets [15]. Response rate should not determine the survey’s quality or bias(es) [42], and additional analyses can be used to help limit bias. For instance, late and non-responder analyses can help researchers determine if their responders aligned with those who chose to opt out or were not initially interested or available to opt in. The response rate has been the long-standing rate used to support sample sizes; however, we suggest that journals, specifically *JAT*, switch to a completion rate. Completion rates consider that individuals use email differently than in the past and may have surveys routed to spam, junk, or quarantine folders. The completion rate is a better representation of those who accessed and completed their participation in the survey rather than using a total population sample that may have been used as a recruitment base.

### Strengths & limitations

The project solely focused on one journal in athletic training with publications over 11 years. Strengths of this project are the comprehensive nature of the data, providing clear trends in published manuscript study designs and sample sizes. The dataset is robust and provides a novel contribution to the literature regarding research methodology. Specifically, the survey research section provides practical benchmarks for the journal editorial board and review team, as well as current and future researchers.

The findings presented from this project may differ if we included other athletic training scholarly outlets, such as the *Journal of Athletic Training Education and Practice (formerly Athletic Training Education Journal)*,* Sports Health*, and *the International Journal for Athletic Therapy and Training.* The project also focused on two metrics – study design and sample size. The project did not extract or analyze power analyses, which the researchers could have used to address the documented sample sizes based on the statistical analyses performed. The rationale for this was that the statistical analysis deployed per study could have impacted the power, meaning each study would require a review of the chosen analysis and the power analysis based on effect size. This could be helpful information for future research to explore. In doing so, we recommend that future research also identify the characteristics of the population studied, as it would glean information on sample sizes specific to target demographics like athletic training students, coaches, or student-athletes. In addition, the research team discovered several inconsistencies in reporting data within the *JAT* publications during data extraction. In many cases, sample sizes and access, response, and completion rates were misreported or not reported. Many abstracts contradicted the data and results in their respective articles, which required additional data extraction and review to verify the correct information.

We recommend future research reviews of published studies within the *JAT* to explore the topic or focus area of the manuscripts. As the research team completed data extraction, we identified a pattern of publishing data specific to some areas of the research agenda, but gaps were noted in other content areas. While this was not the original aim of our project, it is essential to acknowledge the reoccurring topics published in the journal. The NATA’s Research and Education Foundation published the *Athletic Training Research Agenda* (https://www.natafoundation.org/research/atresearchagenda/) to identify and unify research topics of interest to improve athletic training in practice and the profession.

### Recommendations

Each research design and correlating manuscript type offers a distinctive research approach and thus should have different evaluative criteria for the quality of the analysis and presentation of the findings. Regular review and analysis of manuscript types should occur as research in the profession continues to evolve. To guide these decisions, it may be helpful for the *JAT* to report the data of their published materials annually to both the readers and prospective authors to offer data for decision-making and improve overall transparency. We have compiled a list of suggestions for editors, reviewers, authors, and readers of the *JAT* to consider when preparing, reviewing, and reading the literature. These recommendations emerged as areas for continuous quality improvement during the data extraction process or directly from the results. The recommendations in Table 7 are supported by the data and also by discrepancies that were identified in the data extraction process. Dr. Hertel’s vision for the *JAT* in 2018 has become a reality with a marked increase in the impact factor, a noticeable digital footprint, and an increase in thematic issues. As a new era of the *JAT* emerges, we hope that the journal can use the data presented and the recommendations of this study to advocate for continuous quality improvement in the preparation and review of manuscripts. These recommendations require the *JAT* to increase transparency to its potential authors, readers, and reviewers.

**Table 7:** Recommendations

Population of Interest	Recommendations
For Editors and Reviewers	• Ensure alignment of information in the abstract, study design, and results. • All manuscripts should use a pre-determined study design classification found in the *JAT* Author Guidelines. • An official inclusion of mixed-methods and implementation science research guidelines in the *JAT* Author Guidelines. • Add a unique design classification for market items and product design testing. • Only 187 of 1267 published papers over 11 years were survey-based documents within the survey design classification, which can present practical and generalizable data. • Further divide survey-based research into population-based and sample-based • We caution editors and reviewers to require power analysis for survey-based research. • Add a unique design classification for qualitative and epidemiological research involving ATLAS and AT-PRBN programs. • Epidemiological research must state the occurrence of injuries and adverse events rather than the individual patient, if the study allows for it. • Publicly report the rejection rate by research priority area and/or submission type.
For Authors	• Ensure alignment of information in the abstract, study design, and results. • We caution authors to utilize a power analysis when answering research questions that are descriptive in nature. • The integration of a patient-reported outcome measure does not automatically make the study a cross-sectional survey. Survey research requires questionnaires or interviews, from which data can be used and generalized for other populations. • Qualitative research requires open-ended responses or interviews. Using a survey to capture data does not make it qualitative. • Access, completion, and response rates should be included in all survey-based research, if possible. If response rates cannot be calculated in cases such as social media postings and recruitment, this should be noted in the methods, results, and discussion. An exemplar of reporting published in *JAT* is “The Quantitative Examination of the Relationship Between Job Satisfaction and Organizational Fit in Athletic Trainers” [43] • Mixed-methods or multi-methods research should specify whether it is primarily qualitative or quantitative. Additionally, if the study follows a sequential, concurrent, or transformative design, this should be explicitly noted in the paper.
For Readers	• Critically analyze all papers, regardless of sample size, to ensure the data is generalizable and applicable to your clinical question or patient population. • Expand search requests to other design classifications within the *JAT*.

## Conclusion

We identified that the *JAT* continues to trend positively, with an average of 115 manuscripts published annually and mostly published cross-sectional study designs. Survey-based research in the *JAT* has historically exhibited inconsistent reporting practices and expectations in terms of sample sizes and rates for access, response, and completion. The median data presented and definitions for these metrics may be useful in standardizing methodology reporting. Future scholars intending to publish in the *JAT* may benefit from understanding the trends of sample sizes for different manuscript types. Continuous analysis of design, sampling, and reporting strategies should be among the analytics the *JAT* offers as publicly available data about the journal. These metrics provide valuable insights into prevailing sampling practices and standards for data collection and presentation in scholarly articles. In summary, there is a need for greater consistency and transparency in survey-based research reporting. Establishing clear guidelines based on current practices can enhance the quality and reliability of future research contributions to the journal.

## Data Availability

The datasets used and/or analyzed during the current study are available from the corresponding author upon reasonable request.

## Abbreviations

SPSS: Statistical package for social sciences
JAT: Journal of Athletic Training
NATA: National Athletic Trainers’ Association
RCT: Randomized controlled trial
JPE: American journal of pharmaceutical education
IJSM: International journal of sports and exercise medicine
MOST: Multiphase optimization strategy
BOC: Board of certification for the athletic trainer
OMB: Office of management and budget
NSSE: National survey of student engagement
